# Protective immune mechanisms of Yifei Tongluo, a Chinese herb formulation, in the treatment of mycobacterial infection

**DOI:** 10.1371/journal.pone.0203678

**Published:** 2018-09-11

**Authors:** Xin Fan, Ning Li, Xiaoshuang Wang, Jingyu Zhang, Meiyi Xu, Xueting Liu, Beinan Wang

**Affiliations:** 1 CAS Key Laboratory of Pathogenic Microbiology and Immunology, Institute of Microbiology, Chinese Academy of Sciences, Beijing, China; 2 University of Chinese Academy of Sciences, Beijing, China; University of British Columbia, CANADA

## Abstract

Yifei Tongluo (YFTL) is a traditional Chinese medicine (TCM) formulation which has been shown clinical efficacy in treatment of patients with multidrug-resistant tuberculosis in China. However, the underlying mechanisms of the effects of YFTL are lacking. This study investigated the effects of YFTL on immune regulation with a mouse lung infection model with *Bacille Calmette-Guérin* (BCG). We found that compared with untreated mice, the lung mycobacterial load in YFTL-treated mice was significantly reduced, accompanied by alleviated pulmonary inflammation with reduction of pro-inflammatory cytokines and increase of prostaglandin E2 (PGE2). Flow cytometry analyses showed that Th1 cells were significantly higher in the lungs of YFTL-treated mice at early infection time. The results suggest that YFTL-treatment down-regulates pulmonary inflammation, which facilitates a rapid infiltration of Th1 cells into the lungs. Moreover, the Th1 cells in the lungs were resolved faster at later time concomitant with increased the regulatory T cells (Tregs). The reduction of mycobacterial burden associated with improved tissue pathology, faster Th1 cell trafficking, and accelerated resolution of Th1 cells in the lungs of YFTL-treated mice indicates that YFTL improves mycobacterial clearance by maintaining lung homeostasis and dynamically regulating T cells in the lung parenchyma, and suggests that YFTL can be used as host-directed therapies that target immune responses to mycobacterial infection.

## Introduction

*Mycobacterium tuberculosis* (*Mtb*) is a major health problem worldwide that causes mortality of almost 2 million persons per year [1]. The susceptibility to the onset and progression of TB is not only the pathogenic destruction of infected tissue but the outcome of host immune response to the pathogen. Inflammation caused by a wide array of inflammatory cytokines in response to infection dictates the eventual pathology of TB by contributing to early host immune defense and initiating an adaptive immune response, such as activation and recruitment of Th1 cells to the infected site for successful control of TB [2]. However, the excessive inflammation may promote lung tissue damage, which causes TB to spread throughout the lung [3]. Indeed, in *Mtb* infections, disease progression is associated with high levels of pro-inflammatory mediators and progressive accumulation of inflammatory cells in the lungs [4]. Prostaglandin E2 (PGE2) is a lipid mediator and essential homeostatic factor with multiple functions in regulation of immune response [5]. Studies have shown that significantly higher bacterial burden is found in PGE2-deficient MΦs than in wild-type MΦs [6]. Recently, more studies demonstrated that PGE2 production is significantly lower in virulent *Mtb* infection than in avirulent *Mtb* infection and augment of PGE2 levels inhibits *Mtb* replication and prevents acute mortality of *Mtb*-infected mice [7, 8]. These findings indicate that PGE2 is an important mediator in the regulation of immune responses to *Mtb* infection.

Th1 (CD4^+^ IFN-γ^+^) cells in the lung parenchyma are a major element of adaptive immune defense required for containing *Mtb* infections [9]; however, the T-cell response is delayed in *Mtb* infections [10]. It is known that a sufficient recruitment of effective T cells in the lungs requires a homeostatic environment of the lungs and that the inflammatory environment within the lung lesion can limit the acquired immunity [11, 12]. On the other hand, uncontrolled Th1 type immune responses are associated with increased tissue destruction [13, 14]. Therefore, it is important to recruit Th1 cells rapidly to infected sites and to attenuate the process for limiting tissue injury.

Chemotherapy treatment regimens for TB are being complicated by the emergence of multidrug-resistant (MDR) TB [15, 16] and characterized by a long term and complex drug regimen that usually cause problems with toxicity and compliance [17]. Therefore, the development of effective and safe therapies is urgently required. Host-directed therapies (HDTs) that target the host's immune response to the infection can be an efficient treatment for TB [8] and have gained attention as a means to improve treatment outcomes of drug-resistant TB [18]. Yifei Tongluo (YFTL) is a traditional Chinese medicine (TCM) formulation that contains multiple herbs for balance of Yin and Yang, a system of two opposites but complementary aspects of nature to maintain immune homeostasis. Treatment with YFTL with the combination of chemotherapy agents has improved the clinical symptoms of patients with MDR-TB in China [19–21]. However, the mechanisms underlying YFTL treatment of TB have not been investigated although a study shows that YFTL enhances the activities of natural killer cells and balances the ratio of Th1/Th2 cells of immune deficient naïve mice [19]. The purpose of this study is to verify the effects of YFTL on mycobacterial infection and dissect the mechanisms underlying the clinical improvement with a mouse model infected with *Bacillus Calmette-Guérin* (BCG). We found that YFTL treatment significantly reduced mycobacterial numbers in the lungs, associated with restrained early inflammatory responses and alleviated lung tissue damage. In addition, rapider recruitment and faster resolution of Th1 cells in the lungs were detected in the YFTL-treated mice than in the vehicle-treated mice. The results indicate that YFTL reduces mycobacterial burden by balancing innate inflammation and dynamically regulating T cell response in the lungs.

## Materials and methods

### Ethics statement

This study was performed in strict accordance with the recommendations in the Guide for the Care and Use of Laboratory Animals of the IMCAS (Institute of Microbiology, Chinese Academy of Sciences) Ethics Committee. The protocol was approved by the Committee on the Ethics of Animal Experiments of IMCAS (Permit Number: APIMCAS2015010). Mice were bred under specific pathogen-free (SPF) conditions in the laboratory animal facility at IMCAS. All animal experiments were conducted under isoflurane anesthesia, and all efforts were made to minimize suffering.

### Preparation of crude YFTL and conversion of the human dose to the mouse equivalent

The components of YFTL are *Polygonatum sibiricum Delar*, *Bletilla striata*, *Pseudostellariae Radix*, *Stemona sessilifolia*, *Ardisia japonica*, *Viola philippica Cav*, *Cirsii Japonici Herba*, *Asparagus cochinchinensis*, *Flos Farfarae*, *Carapax Trionycis* and *Retinervus Luffae Fructus*. The YFTL extract is provided by Dr. Yanke Liu, Changsha Central Hospital (161 Shaoshan Rd. S, Yuhua Dist, Changsha China) and prepared as described in [19]. The conversion of the human equivalent dose (HED) to the mouse equivalent dose (MED) was calculated according to a previous study by using the following formula for dose translation based on body surface area: HED (mg/kg) = MED (mg/kg) × (Mouse km/Human km), in which mouse km = 3 and human (adult) km = 37. The proposed dose of YFTL was 55.8 μg/mouse (20-g mouse), equivalent to a dose of 0.31 g/kg for human adult per day, according to the previous formula [22].

### Bacterial strains and culture conditions

*Mycobacterium smegmatis (M*. *smegmatis)* strain mc^2^155 and BCG were grown in liquid Middlebrook 7H9 medium (Becton Dickinson) supplemented with 0.2% (v/v) glycerol (Beijing Modern Eastern Fine chemical), 0.05% Tween 80 (v/v) (Sigma) and 10% ADS (50 g/L bovine serum albumin, 20 g/L dextrose and 8.5 g/L NaCl). When required, kanamycin was added at a final concentration of 20 μg/ml. The mc^2^155 and BCG were kindly gifted by Dr. K. Mi (IMCAS). The mc^2^155 and BCG were cultured overnight until the OD_600_ reached 1.7, washed with and resuspended in PBS, and used for all infections. Colony-forming units (CFUs) were verified by plating serial dilutions of cultures onto Middlebrook 7H10 or 7H11 agar plates, followed by 3-day (mc^2^155) or 21-day (BCG) incubations, respectively, at 37°C in 5% CO_2_.

### Mouse model

The mouse infection models were developed as follows: Female C57BL/6 (B6) mice aged 6–8 weeks were randomly divided into two groups (n = 4–6), and the control group was given volumes of H_**2**_O equivalent to the YFTL-treatment volume. Mice were anesthetized with intraperitoneal injection of pentobarbital sodium (60 mg/kg) before inoculation. Mice were intravenous (i.v.) inoculated with 100 μl of *M*. *smegmatis* suspension (1×10^**7**^/mouse) by tail injection, or intranasally inoculated (i.n.) in the left nostril with 50 μl of *M*. *smegmatis* suspension (3.7×10^**7**^/mouse), or 50 μl of BCG suspension (1×10^**7**^/mouse). Animals were placed into cages in a supine position for recovery. Four hours after the inoculation, mice were provided with the YFTL treatment (55.8 μg/mouse) once daily by oral gavage for 7, 14 or 60 consecutive days.

### Specimen collection

Lung tissues were harvested at different time points (3, 5, 7, 14 and 60 days) after infection. The mice were euthanized by CO_2_, and efforts were made to minimize suffering; the lungs were taken, placed in 1 ml of pre-chilled PBS and used for lung tissue preparations and histopathological evaluations. The lung was then pestled and homogenized in 1 ml PBS, passed through a cell strainer (BD Falcon) to obtain a single-cell suspension, and divided into two parts. One part was used for evaluating CFU. The other part was centrifuged for measuring cytokines in the supernatant by enzyme-linked immunosorbent assay (ELISA), and for flow cytometry analysis of cells.

### ELISA assay for cytokines and antibodies

A single-cell suspension of lung was centrifuged at 1,500 rpm for 5 min at 4°C, and IL-1β, TNF-α, IL-6, IL-10, CCL2, IFN-γ, and IL-12p70 levels in the supernatants were measured with ELISA kits (eBioscience, USA) and PGE2 with ELISA kits (MEIBIAO, Beijing, China) according to the manufacturer’s instructions. Briefly, diluted standards and supernatants of lung homogenate were added to 96-well plates precoated with antibodies specific for mouse IL-1β, TNF-α, IL-6, IL-10, CCL2, IFN-γ, IL-12p70 and PGE2. Cytokine binding was detected by enzyme-linked polyclonal antibodies. The intensity was measured at 450/570 nm and the expression levels of cytokines were calculated according to the equation of the standard curve. Sera and supernatants of lung homogenate samples were undiluted and direct dispensed on Corning Costar 96-well plates (Fisher Scientific, Waltham, MA, USA) coated with heat-killed (HK) BCG (1–2×10^7^/well) for testing BCG-specific antibodies. Antibody binding was detected by horseradish peroxidase-conjugated anti-mouse IgG or IgA (Southern Biotech, Birmingham, AL, USA), followed by 3, 3’, 5, 5’-Tetramethylbenzidine staining (Sigma). Absorbance was measured at 450/630 nm. A standard curve was generated by adding two-fold serial dilutions of purified mouse IgG (Alpha Diagnostic Int. Inc., San Antonio, TX, USA) or IgA (Bethyl Laboratories, Montgomery, TX, USA) to anti-mouse IgG- or anti-mouse IgA-coated wells. The concentrations of the immunoglobulin levels were calculated according to the standard curve.

### Isolation of pulmonary mononuclear cells

The pulmonary mononuclear cells were isolated by Percoll (GE Healthcare, catalog no. 17-0891-09, USA) according to the manufacturer’s instructions with modifications. The above single-cell suspension of lung was centrifuged at 1,500 rpm for 5 min at 4°C. After washing with cold D-Hank’s buffer, the cells were isolated by 35% Percoll diluted with D-Hank’s buffer and centrifuged at 2,000 rpm for 15 min at 4°C. The upper liquid phase was removed from the tube; the lymphocyte pellet was resuspended in 0.84% NH_4_Cl solution to lyse the RBCs and then washed twice with cold PBS, stained with antibodies and subjected to flow cytometry analysis.

### Cellular staining and flow cytometry analysis

Cellular staining and flow cytometry analyses for T helper cells and neutrophils were conducted as previously described [23]. Briefly, cells were stained for surface markers with anti-CD4 FITC (clone: GK1.5; eBiosicence, USA), anti-CD8 PerCP (clone: 53–6.7; Biolegend, USA) for T helper cells, and with anti-CD11b FITC (clone: M1/70; Biolegend, USA) and anti-Gr-1 APC (clone: RB6-8C5; Biolegend, USA) for neutrophils. For intracellular staining, fixed cells were permeabilized and stained with anti-IFN-γ APC (clone: XMG1.2; eBioscience, USA). For analysis of Treg cells in the hilar lymph nodes (HLN), single cell suspension of HLN was stained for surface marker with anti-CD4 FITC for 30 min at 4°C in the dark, and washed in cold flow cytometry staining buffer. For intracellular staining, the cells were fixed and permeabilized using cell fixation/permeabilization kit (eBiosicence, catalog no. 00-3525-00, USA), and incubated with anti-Foxp3 APC (clone: Fgk-16s, eBiosicence, USA) for 60 min in the dark at 4°C. Cells were washed with 1 ml of permeabilization buffer and resuspended in staining buffer for analysis. Samples were analyzed using a FACSCalibur or FACSCanto flow cytometer (BD Biosciences) and FlowJo sofware (Treestar, Ashland, OR, USA).

### MTT assay

Cytotoxicity of splenocytes was measured using the 3- (4, 5-dimethylthiazol-2-yl) -2, 5 -diphenyltetrazolium bromide (MTT) assay. The splenocytes were suspended in complete RPMI-1640 medium (Invitrogen, Carlsbad, CA, USA) containing 10% FCS, 2 mM L-glutamine, 100 U/ml penicillin G and 100 mg/ml streptomycin at a density of 5×10^5^ cells/ml in 96-well plates, and cultured in the presence or absence of YTFL (300 μg final concentration) at 37°C in 5% CO_2_. After 24 hr incubation 20 μl of the MTT solution (5 mg/L) was added to each well and reincubated for four hours at 37°C in 5% CO_2_ before the medium was discarded. Then, 150 μl of dimethyl sulfoxide (DMSO) was added to dissolve the formazan crystals. The plate was shaken for 30 min to dissolve the crystals and the absorbance was measured at 490 nm with a microplate reader [24].

### Lymphocyte co-culture with bone marrow-derived dendritic cells

Bone marrow-derived DCs (BMDCs) were generated from eight to ten-week old C57BL/6 (B6) mice according to a previously described protocol [25]. Briefly, erythrocyte-depleted murine bone marrow cells from femurs and tibias were cultured in complete RPMI 1640 medium with mouse GM-CSF (20 ng/ml) (Peprotech, Rocky Hill, USA). On day six, more than 90% of the cultured cells were determined to be DCs, which displayed a dendritic shape. These BMDCs were used for co-culture experiments. The BMDCs (1×10^5^cells/well) were stimulated with HK-BCG (MOI = 1:10) and cultured in complete RPMI 1640 in 24-well plates at 37°C in 5% CO_2_ for 16–24 hr. The stimulated BMDCs were co-cultured with splenocytes (1×10^6^ cells/well) from naïve mice in the presence or absence of YFTL, and incubated at 37°C in 5% CO_2_ for three days. The culture supernatants were collected for detection of the expression levels of IFN-γ and IL-12p70 by ELISA.

### Statistical analysis

Each experiment was performed at least twice with 3–6 mice or samples per group. The lung weights and the number of neutrophils in the lungs were analyzed by One-way ANOVA followed by Bonferroni’s correction. CFUs were analyzed by two-tailed unpaired Mann-Whitney *U* nonparametric *t* tests, and two-tailed unpaired *t* test for cytokine and others using GraphPad Prism (Version 6.0 for Windows; GraphPad Software). The data were considered significantly different at *P* < 0.05.

## Results

### YFTL reduced the mycobacterial load in the lungs of mice infected with *M*. *smegmatis* or BCG

**Fig 1:**
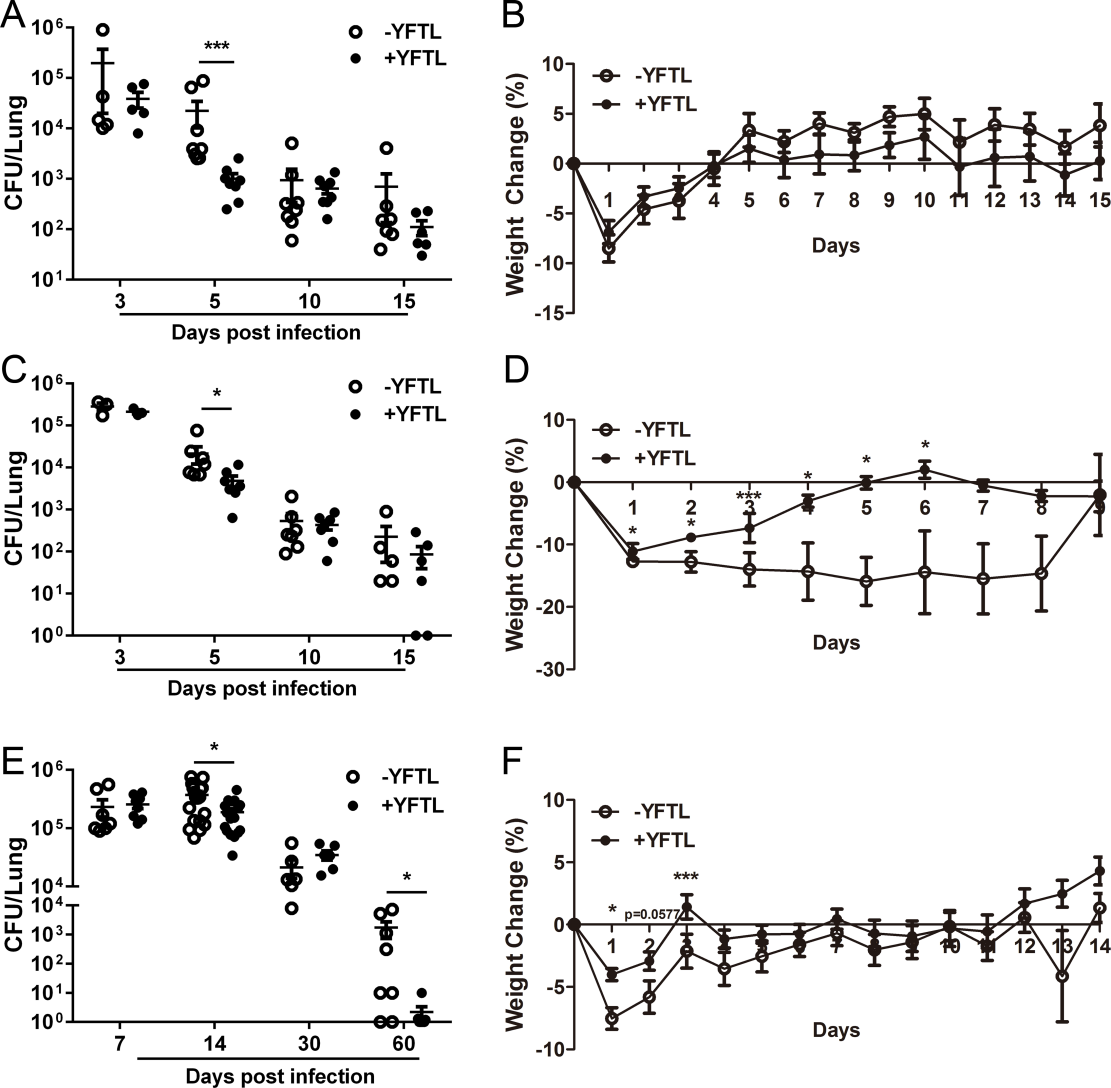
YFTL treatment increases therapeutic efficacy against Mycobacterial infections in mouse models. Mice were i.v. inoculated with 100 μl of *M*. *smegmatis* suspension (1×10^7^/mouse) by tail injection (A and B) or i.n. inoculated with 50 μl of *M*. *smegmatis* suspension (3.7×10^7^/mouse) into the left nostril (C and D). For BCG infection model mice were i.n. inoculated with 50 μl of BCG suspension (1×10^7^/mouse) (E and F). Four hours after the inoculation, mice were treated once daily with YFTL, and the control group was given an equal volume of H_2_O by oral gavage. (A, C, and E) Lung tissues were harvested and homogenized for CFU counting at indicated times after the inoculation. The data represent the geometric means of two independent experiments (n = 6–8) and were analyzed with Unpaired Mann-Whitney *U* nonparametric *t* tests. (B, D, and F) The weight of the mice was measured once daily. * *P* < 0.05, *** *P* < 0.001.

We first evaluated the effects of YFTL on mycobacterial infection using an *M*. *smegmatis* infection model by intravenous injection (i.v.) [26]. Mice were infected (i.v.) with the *M*. *smegmatis* strain mc^2^155. Four hours later one group of mice were given YFTL and the other (untreated) were given a volume of water equivalent to that used for the YFTL dose once daily by oral gavage. Bacterial colonization was determined by colony-forming units (CFUs) in the lungs and body weight was monitored daily. The results showed that CFU numbers from the lungs of YFTL-treated mice was significantly lower than that in the untreated mice at five days post infection (p.i.), although was no difference between -YFTL and +YFTL group at other time points (Fig 1A). No significant differences in body weight were observed between the YFTL-treated and untreated mice (Fig 1B).

Mtb inhalation is the main route of infection that causes TB. We examined the effects of YFTL in a model of intranasal (i.n.) infection. Mice were infected i.n. with mc^2^155 and treated with YFTL according to the protocol from the i.v. infection model. Similarly, at five days p.i., the number of CFUs recovered from the lungs of YFTL-treated mice was significantly lower than that in the untreated mice, although no differences in CFUs were found at other time points (Fig 1C). Interestingly, compared with the i.v. infection method, body weight recovery was significantly faster in the YFTL-treated mice (Fig 1D), indicating that the i.n. infection model is more sensitive to YFTL treatment. In summary, these results suggest that YFTL treatment alleviated the symptoms of the infection and temporarily restricted bacterial replication in the lungs.

We further tested the effects of YFTL on *Bacille Calmette-Guérin* (BCG) infection model with i.n. inoculation, a more sensitive model for mycobacterial studies [27]. Mice were infected i.n. with BCG and treated with YFTL according to the protocol used for the *M*. *smegmatis* infection. As shown in Fig 1E, the number of CFUs in the lungs of treated and untreated group was similar at seven days p.i., but at 14 days p.i., the number of CFUs in the YFTL-treated mice was significantly lower than in the untreated mice. At 60 days p.i., more than 1**×**10^3^ CFUs were detected in the untreated mice, whereas the bacteria were almost cleared completely in the YFTL-treated mice; although the number of CFUs at 30 days p.i. was similar in both groups. However, the number of CFUs in the spleens at the indicated time points or spleen weights at 14 days p.i. between YFTL-treated and untreated groups were not significantly changed (S1 Fig). Body weight loss in the YFTL-treated mice was slightly less severe than in the untreated mice, with significance at three days p.i.. Both groups of mice had returned to their original body weight by 14 days p.i. (Fig 1F). These results confirmed that YFTL treatment can reduce mycobacterial burden in the lungs. The BCG model was used in following experiments to study effects of YFTL on immune response to mycobacterial infection.

### YFTL treatment significantly reduces pulmonary pathogenesis

**Fig 2:**
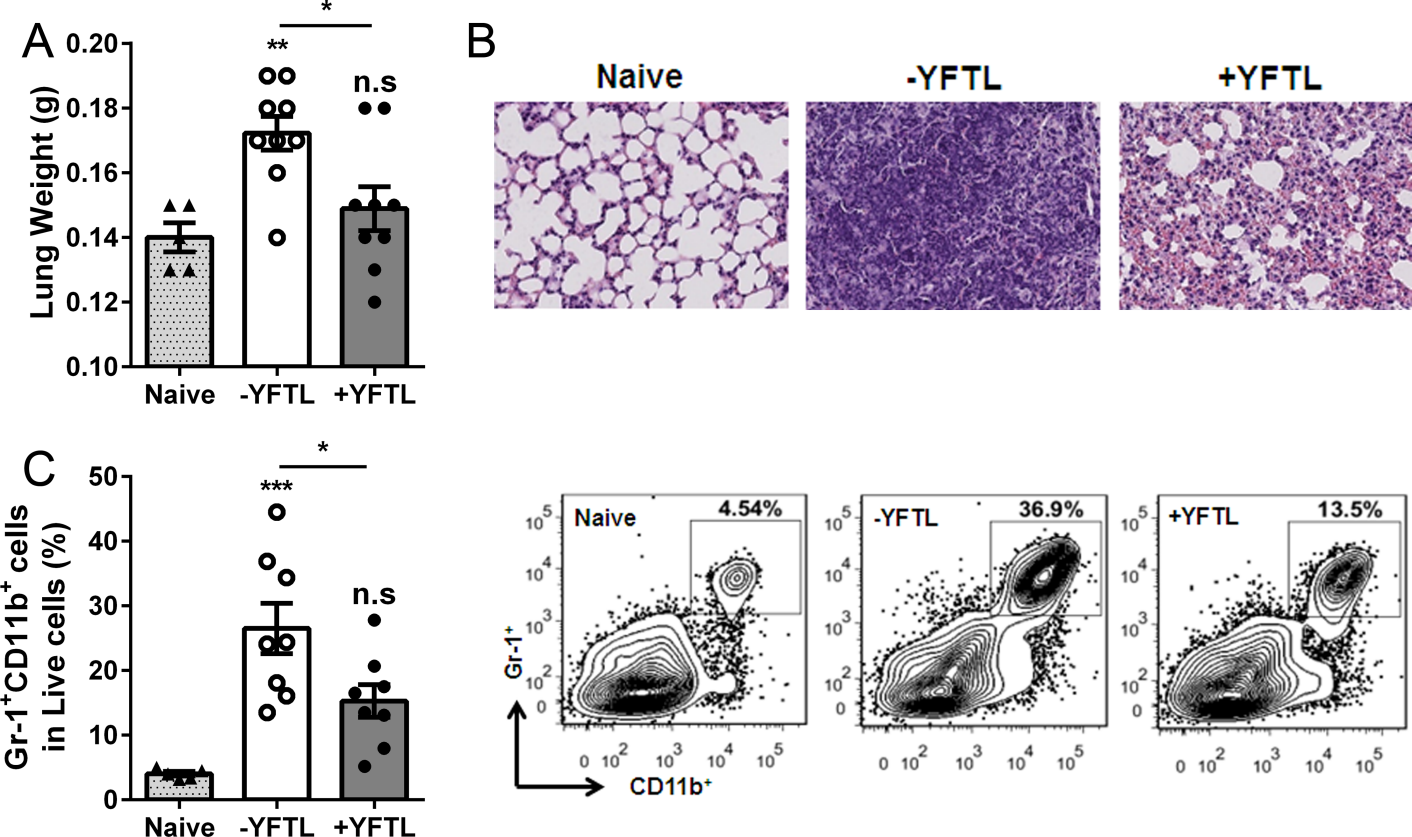
Less pulmonary pathology was observed in YFTL-treated mice. (A) The lungs were weighed at three days p.i. and (B) The histopathology of the lungs was assessed in sections stained with hematoxylin and eosin (magnification × 20). (C) Lung monocytes were stained with FITC-CD11b and APC-Gr-1 and analyzed by flow cytometry. Results are presented as means ± SEM from two independent experiments. Statistical analysis by one-way ANOVA with Bonferroni post tests. * *P* < 0.05, ** *P* < 0.01, *** *P* < 0.001, n.s., not significant. Representative flow cytometry plots are shown on the right.

Because YFTL does not have bactericidal activity *in vitro* we predicted that the reduced CFUs in the YFTL-treated mice could be resulted from host response to the infection. At three days p.i. when the body weights were significantly different between the treated and untreated groups, the lung weights of the untreated mice were substantially increased; whereas, no significant difference was found in the YFTL-treated mice compare with naïve mice (Fig 2A). Histological examination of the lung tissue sections showed that the BCG infection caused significant solidification of the lungs; however, while the untreated mice showed few intact alveolar spaces and a heavy infiltration of inflammatory cells (Fig 2B, center), there was much more alveolar space and less infiltration of inflammatory cells in the lungs of YFTL-treated mice (Fig 2B, right). Consistently, flow cytometry analyses revealed that the number of neutrophils in the lungs was significantly lower in the YFTL-treated mice than in the untreated mice (Fig 2C), although was no difference between naïve and +YFTL groups. These findings indicate that YFTL alleviated the pulmonary pathology by reducing the inflammatory responses to the mycobacterial infection.

### YFTL treatment significantly reduced the production of inflammatory cytokines in the lungs of BCG-infected mice

**Fig 3:**
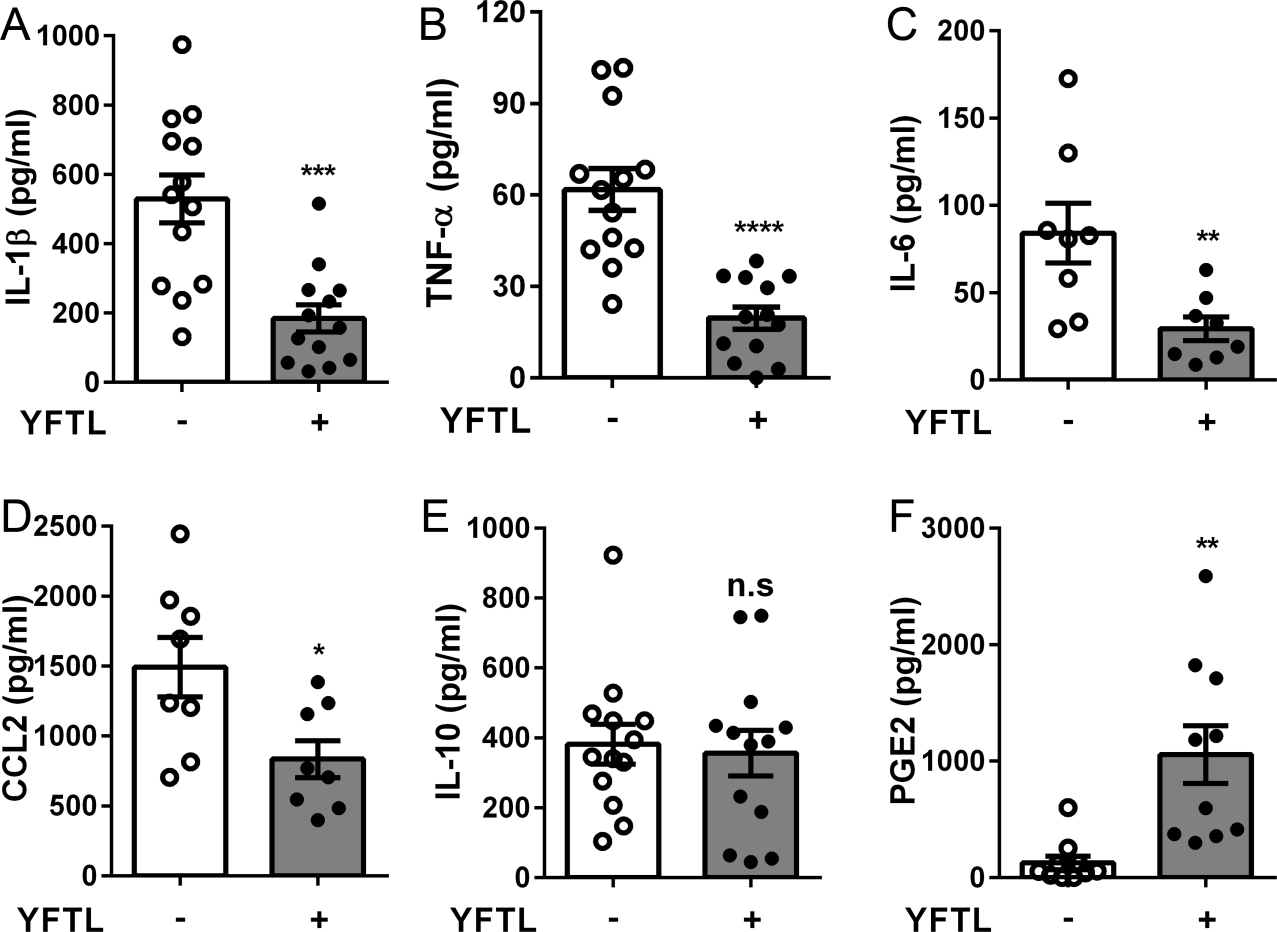
YFTL treatment significantly alleviated inflammatory response in the lungs of infected mice. (A-F) The expression levels of IL-1β, TNF-α, IL-6, CCL2, IL-10 and PGE2 in the supernatants of lung homogenates of mice at therr days p.i., as determined by ELISA. Data are presented as means ± SEM from two or three independent experiments. * *P* < 0.05, ** *P* < 0.01, *** *P* < 0.001, **** *P* < 0.0001, n.s., not significant.

We speculated that the alleviated pulmonary pathology in YFTL-treated mice results from reduced expression of pro-inflammatory cytokines. ELISA examination of the supernatants of lung tissue homogenate showed that the levels of IL-1β, TNF-α and IL-6 were lower in the YFTL-treated than in the untreated mice at three days p.i. (Fig 3A–3C). Less infiltration of inflammatory cells in the lungs of YFTL-treated mice suggests that YFTL may reduce chemokine production. It has been reported that a high level of CCL2 (chemokine ligand 2) is associated with disease severity in TB patients [28]. We examined CCL2 by ELISA and results showed that CCL2 levels in the lungs of YFTL-treated mice were lower than in the untreated mice (Fig 3D). These results suggest that the reduction in pro-inflammatory mediators diminishes the recruitment of inflammatory cells and alleviates inflammation in the lungs.

A balanced inflammation is required for host protection [29] and maintained by anti-inflammatory cytokines, such as IL-10. However, ELISA tests for detection of IL-10 showed that IL-10 production in the lungs was similar between the YFTL-treated and untreated mice at three days p.i. (Fig 3E), indicating that IL-10 is not involved in the YFTL-mediated inhibition of early lung inflammation. Prostaglandin E2 (PGE2) is another mediator that is able to prevent uncontrolled inflammation [30]. We found that PGE2 increased in response to BCG infection at three days p.i.; but dramatically higher levels of PGE2 were detected in the YFTL-treated mice than in the untreated mice (Fig 3F). The association of alleviated lung inflammation with increased expression of PGE2 indicates that YFTL balanced pulmonary inflammation by induction of PGE2. The results also suggest that a controlled lung inflammatory response to *Mtb* during early infection is important in the pathology of mycobacterial infection.

### YFTL treatment changed the dynamics of Th1 cells in the lungs but had no effect on antibody responses to mycobacterial infection

**Fig 4:**
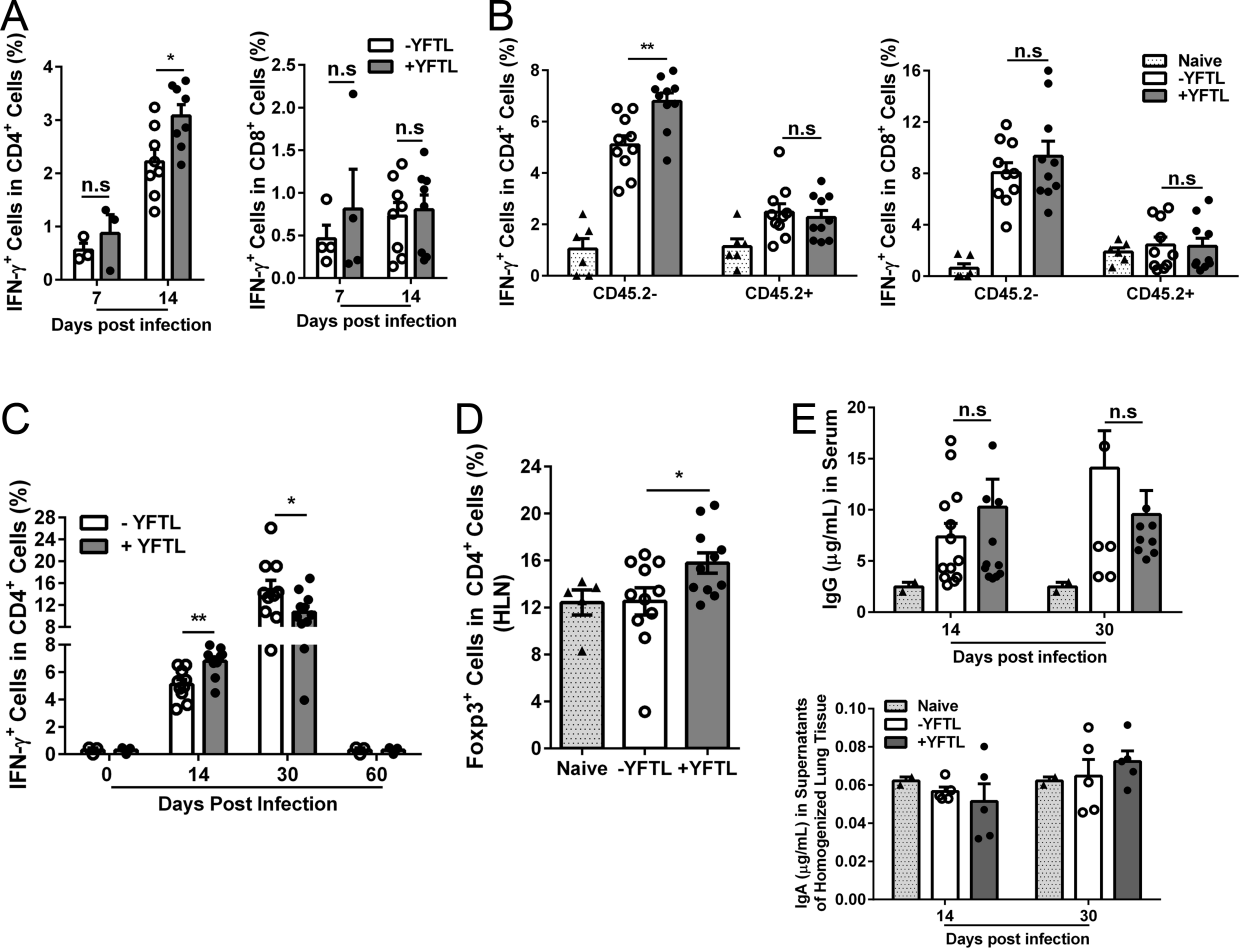
YFTL treatment increased the number of CD4^+^ IFN-γ^+^ cells in the lung parenchyma. (A) Number of CD4^+^ IFN-γ^+^ cells (left) and CD8^+^ IFN-γ^+^ cells (right) were determined by flow cytometry at 7 and 14 days p.i.. (B) Lung parenchymal-localized Th1 cells (CD4^+^ IFN-γ^+^ CD45.2^-^) (left) and CTL cells (CD8^+^ IFN-γ^+^ CD45.2^-^) (right) were stained and analyzed by flow cytometry at 14 days p.i. or (C) at different times p.i. (D) Single-cell suspensions were prepared from HLN of mice at 30 days p.i. then stained for Treg cells (CD4^+^ Foxp3^+^) and analyzed by flow cytometry. (E) The BCG-specific IgG in the blood (upper panel) and IgA in the supernatants of homogenized lung tissue (lower panel) were determined by ELISA at 14 and 30 days p.i.. Data are presented as means ± SEM from two to three independent experiments, * *P* < 0.05, ** *P* <0.01, n.s., not significant.

To determine whether the YFTL-mediated resistance to the BCG infection was related to the T cell response, single-cell suspensions of homogenized lung tissue were analyzed by flow cytometry. We found that Th1 cells (CD4^+^ IFN-γ^+^) were increased in response to BCG infection at 7 and 14 days p.i.. Noticeably, the number of these cells was significantly higher in the YFTL-treated mice than in the untreated mice at 14 days p.i. (Fig 4A, left). CTL cells (CD8^+^ IFN-γ^+^) were also increased in the lungs at 14 days p.i. with no difference between the YFTL-treated and untreated groups (Fig 4A, right). Recent studies have shown that lung parenchymal T cells exhibit a greater control over *Mtb* infections than intravascular T cells [9]. To determine whether the increased Th1 cells reflect those cells in the lung parenchyma, intravascular staining was used to discriminate between tissue-localized and blood-borne cells in the lungs [31]. Flow cytometry analyses showed that at 14 days p.i. the number of Th1 cells in the lung parenchyma of YFTL-treated mice was higher than in the untreated mice (Fig 4B, left), but the number of these cells retained within the lung blood vasculature was comparable between the YFTL-treated and untreated mice. In contrast, the number of CTL cells was similar between the two groups in both the parenchyma and the blood vasculature (Fig 4B, right). Taken together these results indicate that YFTL treatment promotes a rapid infiltration of Th1 cells, but not CTL cells, into the lung parenchyma.

The Th1 cells in the lung parenchyma were continuously increased to higher levels at 30 days p.i. after the infection with and without YFTL treatment; however, lower levels of these cells were found in the YFTL-treated mice than in the untreated mice (Fig 4C) with similar CFU numbers in the lungs between the two groups at this time point (Fig 1E). By 60 days p.i., the Th1 cells returned to basal levels in both YFTL-treated and untreated groups (Fig 4C) when the bacteria in the lungs were completely eradicated in seven of the eight YFTL-treated mice (87.5%) but were eradicated in only two of the eight untreated mice (25%) (Fig 1E). These results suggest that YFTL treatment results in a timely resolution of the Th1 response in the lungs, which favors clearance of the bacteria. To determine if the lower Th1 response was related to the Treg cell response in YFTL-treated mice at 30 days p.i., the number of CD4^+^ Foxp3^+^ T cells was determined. We found that CD4^+^ Foxp3^+^ T cells were hardly detectable in lung parenchyma (data not shown). However, the number of these cells was increased in the hilar lymph nodes (HLN) of YFTL-treated mice, compared with that of the untreated mice (Fig 4D), suggesting that the rapid Th1 resolution in YFTL-treated mice is related to the increase in Treg cell numbers.

In contrast to the effects of YFTL on T cell-mediated immunity, BCG-specific antibodies determined by ELISA showed that serum IgG was significantly increased in response to BCG infection (Fig 4E, upper panel), but no differences were found between the YFTL-treated and untreated mice at the tested time points. Homogenized lung tissue were also measured for IgA and showed that no BCG-specific IgA response was detected in all groups of mice (Fig 4E, lower panel). Taken together the results indicate that YFTL treatment mediates resistance to mycobacterial infection through regulation of Th1 cells but not antibodies.

### YFTL promoted activation of splenocytes in vitro

**Fig 5:**
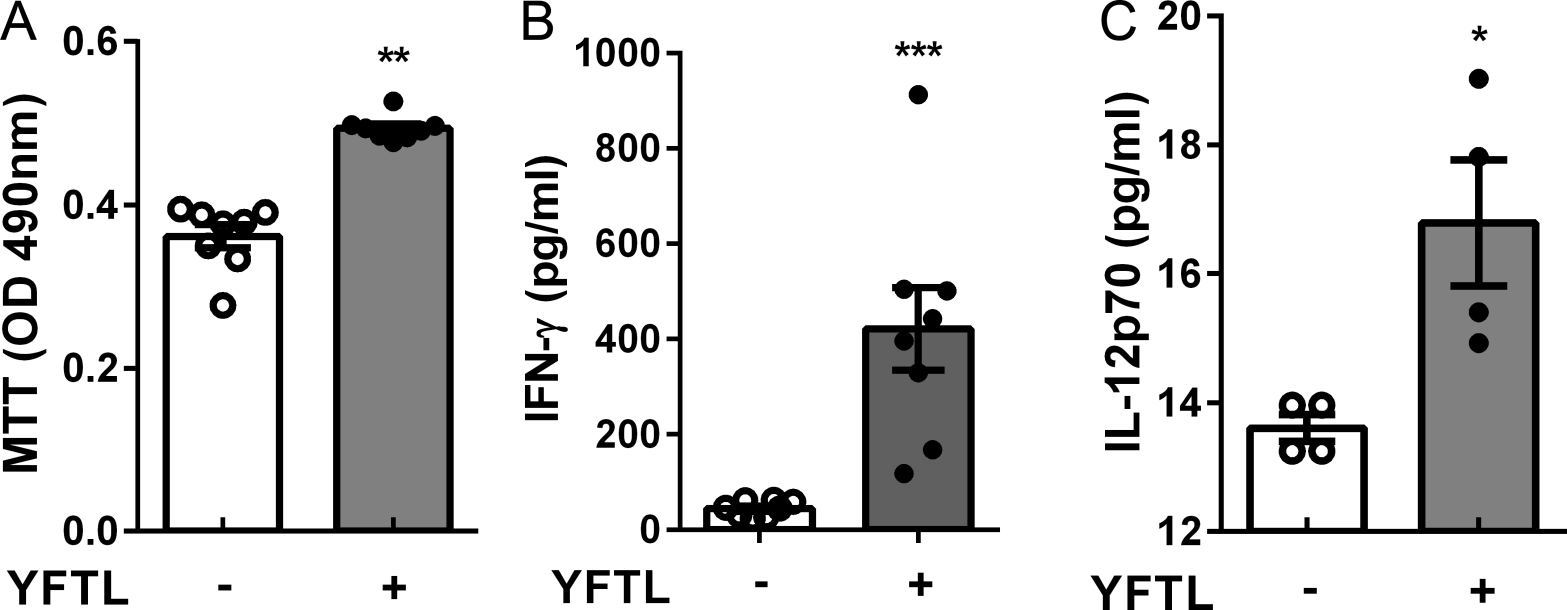
YFTL promoted activation of splenocytes *in vitro*. (A) Splenocytes were incubated in the presence or absence of YTFL for 24 hr. Cytotoxicity of splenocytes was assessed using MTT method. (B and C) BMDCs were pretreated with HK-BCG for 16–24 hr and co-cultured with splenocytes from naïve mice in the presence or absence of YFTL for three days. The expression levels of IL-12p70 and IFN-ɣ in the culture supernatants were determined by ELISA. Data are representative of two independent experiments and presented as means ± SEM.* *P* < 0.05, ** *P* <0.01, *** *P* < 0.001.

The rapid early increase of Th1 cells in the lung parenchyma could be caused by improved T cell activation. *In vitro* assays were performed to determine if YFTL can promote lymphocyte activation. Naïve splenocytes were pretreated with YFTL or medium alone, and the cells were examined by MTT assay for viability. A higher viability was found in YFTL-treated than -non-treated splenocytes (Fig 5A). Then the same numbers of live splenocytes treated with or without YFTL were co-cultured with BCG-primed BMDCs, respectively. Cytokine production in the culture supernatants was determined by ELISA. As showed in Fig 5B and 5C higher production of IFN-ɣ and IL-12p70 was observed in the supernatant of the cultures treated with YFTL, indicating that YFTL directly promoted Th1 response to mycobacteria.

## Discussion

Susceptibility to mycobacteria can result from either inadequate or excessive acute inflammation [32]. The immune response to *Mtb* must be tightly regulated to mount a sufficient response to contain bacterial growth while avoiding inflammation-mediated tissue damage. In the study, we employed a BCG infection model and demonstrate that YFTL, a clinically effective TCM formulation used in the treatment of TB patients, improves outcomes of BCG infected mice by regulating host immune responses, leading to increased eradication of the bacteria. The results reveal that the beneficial effects of YFTL on mycobacterial infection are due to its ability to suppress early inflammation in the lungs through PGE2 production and to regulate the dynamics of Th1 cell numbers in the lung parenchyma.

PGE2 has multiple functions in regulation of immune response, including suppression of acute-inflammatory mediators [5]. It is reported that PGE2 induced by BCG is involved in the Th1-to-Th2 shift of immune responses [33, 34], which is not favor clearance of mycobacterial infection. Recently, more studies demonstrated the critical role of PGE2 in inhibition of *Mtb* replication. However, the mechanisms are not clear. We found that YFTL significantly promoted PGE2 production in the lungs, concomitant with restrained inflammatory cytokines and alleviated pathology. These results indicate that increased endogenous PGE2 contributes to the lung homeostasis and suggest that the role of PGE2 might be stage-dependent, which is beneficial at early time of BCG infection by maintenance of innate immune response and detrimental to adaptive immune response by interfering with Th1 response. YFTL could avert destructive tuberculous lung pathology through induction of PGE2 at early time. More investigation is required to study the mechanisms underlying the regulation of PGE2 and dynamics of PGE2 production during YFTLtreatment.

The coordinated efforts of innate and adaptive immune responses are required for the effective host resistance to *Mtb* infection [11]. Th1 response is induced by BCG and plays important role in clearance of BCG in the lungs of mice [35, 36]. Higher levels of Th1 cells at early time in YFTL-treated mice could be due to YFTL-improved lung homeostasis during innate inflammatory response to the infection, which benefits trafficking of Th1 cells to the infected sites and contributes to decreasing the lung bacterial burden during this lag time between the establishment of infection and the arrival of T cells. The alveolar macrophage (AMΦ) plays a key role in the immediate elimination of the bacteria by phagocytosis and is important in both the induction and effector phases of specific T cell responses by its multiple functions [37, 38]. However, we found that the expression of *iNOS* mRNA was induced following infection of mice with BCG and was significantly suppressed by YFTL treatment at three days p.i. (data not shown). It has been reported that T cell proliferation is suppressed significantly by AMΦs [39]. Therefore, it is possible that YFTL-mediated reduction of iNOS is part of dampened lung inflammation and beneficial to T cell activation. In addition, *in vitro* induction of splenocytes activation and production of IFN-γ and IL-12p70 in these cells by YFTL also supports a contribution of direct role of YFTL in activation of Th1 cells. The effects of YFTL on multiple functions of AMΦs in both innate and adaptive immune responses will be investigated in our future study.

Treg cells are required for immune homeostasis and constitute an essential counterbalance of inflammatory Th1 responses. Although emerged studies point out the role of Treg in determining severity of TB, the function of Treg in TB still remains obscure and studies on the role of Treg cells in BCG infection are rare. It is found that a strong Treg cell response is associated with less effective BCG vaccination [40], indicating that Treg cells are indicative of ineffective immune response induced by BCG. While a number of studies support that Treg cells are associated with a detrimental outcome of active TB other studies demonstrate Treg cells are correlated with protection [41, 42]. These differences may be dependent on the stage of an infection when the samples are taken for examination. We found that increased Treg cells in the HLN of YFTL-treated mice at 30 days p.i., concomitant with faster resolution of Th1 cells and more effective bacterial clearance subsequently; indicating that the Treg cells contribute to damping of Th1 response and suggesting that differential regulation of Th1 response is important for the outcome of mycobacterial infection.

A high structural diversity of compounds is expected in YFTL. These components may act to improve the therapeutic effects synergistically or independently. As a starting point to study mechanisms underlying YFTL-mediated improvement in TB treatment we focused on the role of YFTL as a formula in immune response to mycobacterial infection. The phytochemical active ingredients in YFTL, their metabolites *in vivo* and the cellular mechanisms involved in the modulation of immune responses remain to be further elucidated to improve clinical results of YFTL. TCM focuses on holistic regulation of immune response [43]. Although the pharmacologically active ingredients in YFTL remain to be further elucidated we showed that YFTL supported a comprehensive immune functioning by dynamically regulating immune response to mycobacterial infection. The study indicates that a coordinated and dynamic immune regulation is critical for a beneficial outcome of TB. Most HDTs are still theoretical so far, except for the use of broadly acting corticosteroids, which may cause immunosuppression, leading to insufficient immunity against TB [44]. Thus, more specific host-directed therapies are desired. The effects of YFTL on enhancement of PGE2 production and regulation of Th1 cells may make YFTL a HDT in TB treatment.

The findings in the study encourage us to ask more questions: 1) what is the efficacy of YFTL in a mouse model infected with a pathogenic *Mtb* strain? 2) If the infection will start again after 60 days of YFTL treatment is interrupted? 3) If YFTL promotes macrophage apoptosis and bacterial killing by macrophages with increased PGE2? In addition, refining YFTL formulation for more desired anti-TB effects would further improve the efficacy of YFTL in TB treatment and provide an effective alternative treatment for MDR *Mtb*.

## Supporting information

S1 Fig The number of CFUs in the spleens were similar between the YFTL-treated and untreated mice. Mice were i.n. inoculated with BCG (1×10^7^ CFU/50 μl/mouse). (A) Spleen tissues were harvested and homogenized for CFU counting 14, 30, and 60 days after the inoculation. (B) The spleens were weighed at 14 days p.i.. Data are from one-two independent experiments and are presented as means ± SEM. * P < 0.05, ** P < 0.01, n.s., not significant.
(TIF)
