# Supplementary figures and images for: Protective immune mechanisms of Yifei Tongluo, a Chinese herb formulation, in the treatment of mycobacterial infection
Source: PLoS One. 2018 Sep 11;13(9):e0203678. doi: 10.1371/journal.pone.0203678 (PMC6133367; doi:10.1371/journal.pone.0203678)

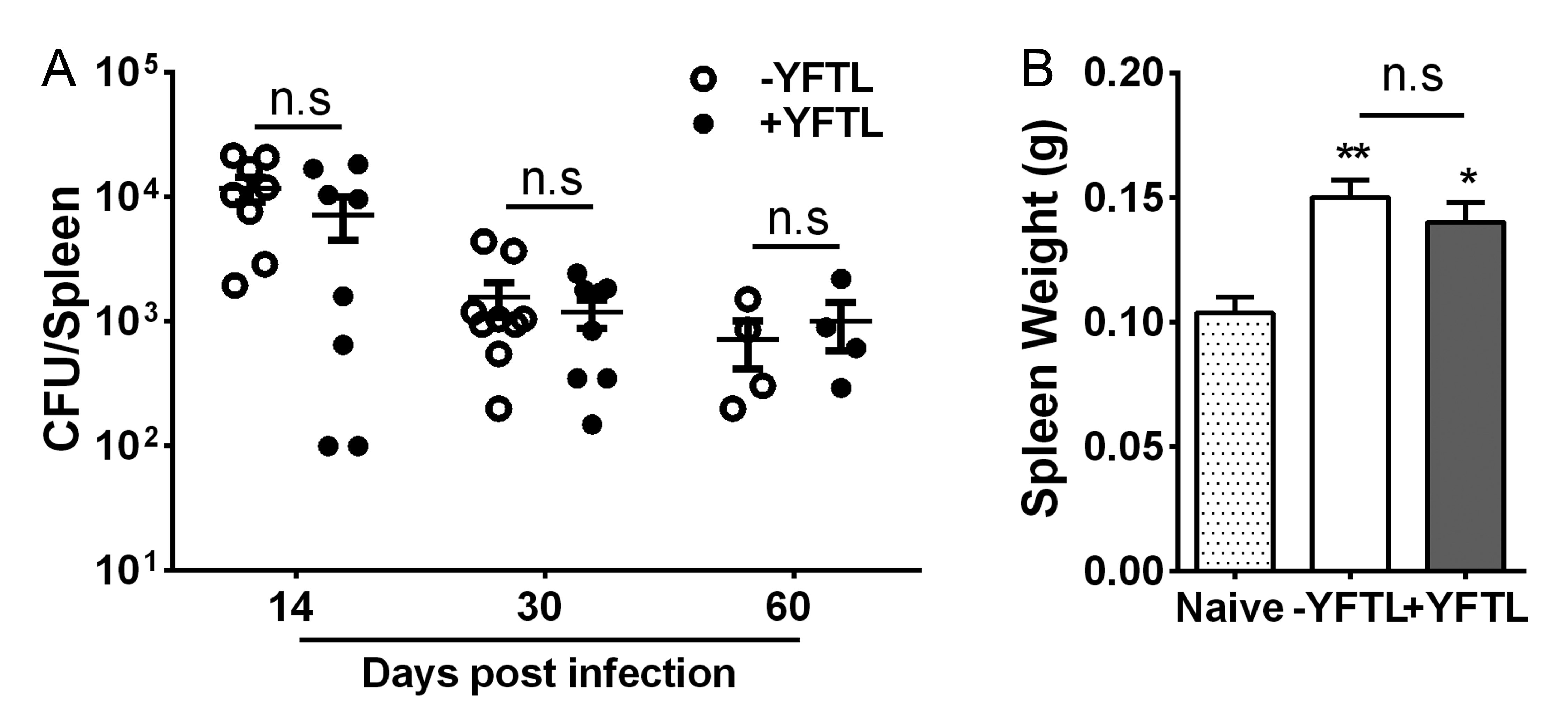

Supplement: S1 Fig — Mice were i.n. inoculated with BCG (1×10^7^ CFU/50 μl/mouse). (A) Spleen tissues were harvested and homogenized for CFU counting 14, 30, and 60 days after the inoculation. (B) The spleens were weighed at 14 days p.i.. Data are from one-two independent experiments and are presented as means ± SEM. * P < 0.05, ** P < 0.01, n.s., not significant. (TIF) [file pone.0203678.s001.tif]
